# Microbe-cellulose hydrogels as a model system for particulate carbon degradation in soil aggregates

**DOI:** 10.1093/ismeco/ycae068

**Published:** 2024-05-04

**Authors:** Pieter Candry, Bruce J Godfrey, Mari Karoliina-Henriikka Winkler

**Affiliations:** Civil and Environmental Engineering, University of Washington, 201 More Hall, Seattle, WA 98195-2700, United States; Laboratory of Systems and Synthetic Biology, Wageningen University & Research, 6708 WE, Wageningen, The Netherlands; Laboratory of Systems and Synthetic Biology, Wageningen University & Research, 6708 WE, Wageningen, The Netherlands. E-mail: pieter.candry@wur.nl; Civil and Environmental Engineering, University of Washington, 201 More Hall, Seattle, WA 98195-2700, United States; Civil and Environmental Engineering, University of Washington, 201 More Hall, Seattle, WA 98195-2700, United States

**Keywords:** experimental model, carbon cycling, soil microbiology

## Abstract

Particulate carbon (C) degradation in soils is a critical process in the global C cycle governing greenhouse gas fluxes and C storage. Millimeter-scale soil aggregates impose strong controls on particulate C degradation by inducing chemical gradients of e.g. oxygen, as well as limiting microbial mobility in pore structures. To date, experimental models of soil aggregates have incorporated porosity and chemical gradients but not particulate C. Here, we demonstrate a proof-of-concept encapsulating microbial cells and particulate C substrates in hydrogel matrices as a novel experimental model for soil aggregates. *Ruminiclostridium cellulolyticum* was co-encapsulated with cellulose in millimeter-scale polyethyleneglycol-dimethacrylate (PEGDMA) hydrogel beads. Microbial activity was delayed in hydrogel-encapsulated conditions, with cellulose degradation and fermentation activity being observed after 13 days of incubation. Unexpectedly, hydrogel encapsulation shifted product formation of *R. cellulolyticum* from an ethanol-lactate-acetate mixture to an acetate-dominated product profile. Fluorescence microscopy enabled simultaneous visualization of the PEGDMA matrix, cellulose particles, and individual cells in the matrix, demonstrating growth on cellulose particles during incubation. Together, these microbe-cellulose-PEGDMA hydrogels present a novel, reproducible experimental soil surrogate to connect single cells to process outcomes at the scale of soil aggregates and ecosystems.

## Graphical Abstract

**Graphical Abstract:**
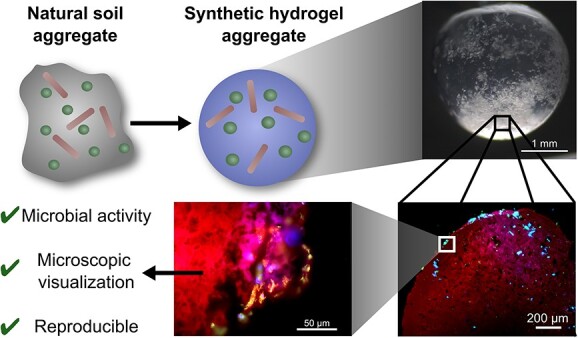

## Introduction

Microbial degradation of particulate carbon (C) governs C cycling in environmental settings [1]. Microbes depolymerize particulate C in soils from e.g. leaf litter into its chemical building block constituents (i.e. sugars, peptides, fats) before further converting this C to CO_2_ or—under anaerobic conditions—CH_4_, while a fraction of the C will be retained and potentially stored on the long-term as soil organic matter. The release and retention of C makes soils net sinks or sources of greenhouse gases, imparting a significant impact of particulate C degradation on global climate processes [2, 3]. In parallel, microbial degradation, release, and retention of C also affects soil health and food productivity in agricultural contexts [4]. While the micrometer-scale microbial mechanisms and global-scale impacts of particulate C degradation are well described, how *in situ* factors such as biofilms, aggregate structures and pores, and soil architecture connect processes across these scales remains poorly understood [5, 6].

Dedicated experimental methodologies are required to investigate microbial particulate C degradation in soils. Investigating microbial communities in complex soil matrices—either *in situ* (i.e. natural soils) or *ex situ* (i.e. soil incubations)—can inform the role of spatial niches and microbe-mineral interactions in soil C storage [1, 7, 8]. Yet, soils are chemically, spatially, and microbially highly complex [4, 7, 9], hindering mechanistic studies connecting microbial functions and activities to ecosystem outcomes [6]. Enriching and isolating particulate C degrading communities and organisms in laboratory settings is an alternative approach that can identify C cycling organisms and their physiology. However, removing microbes from their environmental context ignores their connection to spatial processes such as diffusion gradients in soil aggregates [4, 10]. Fabricated ecosystems are experimental models designed to bridge these two approaches, combining the reproducibility of well-known microbes with ecosystem-specific processes in a highly controllable experimental system [11]. Microfluidics chips replicating soil architecture and soil aggregate chemical gradients have been used to elucidate how microbial community structure and function is governed by diffusion gradients and cell dispersal in pore networks [12–14]. Despite the strengths of these spatially explicit experimental models, particulate C degradation has so far not been incorporated.

**Figure 1:**
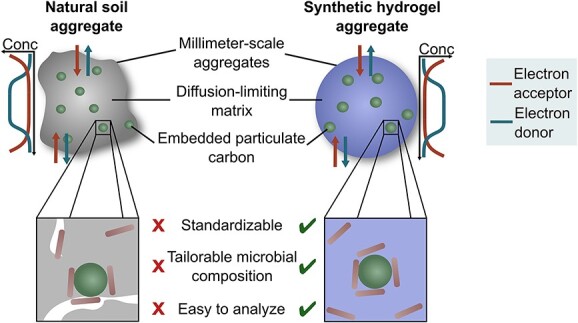
Hydrogel-based synthetic soil aggregates. A schematic representation of key soil aggregate processes that can be reproduced by hydrogel-based aggregates, as well as the key benefits that could be delivered by the hydrogel platform.

Hydrogels offer a powerful alternative to existing microbe-soil aggregate experimental models [15] (Fig. 1). Hydrogels are water-retaining polymers that can be assembled into spherical, millimeter-scale particles similar in size to soil aggregates with several key features enabling their use as experimental models [16, 17]. First, hydrogel matrices limit diffusion of nutrients from the aqueous phase inward and can replicate chemical gradients (e.g. O_2_) present in biofilms and soil aggregates [18–21]. Second, hydrogels as synthetic soil aggregates allow the use of either pure cultures, synthetic communities, native soil communities, and any combination thereof depending on the question at hand. Third, hydrogel matrices are chemically simple and mechanically robust, facilitating downstream molecular and microscopic analyses [21, 22]. However, care should be taken in selecting suitable hydrogel matrices; commonly used alginate-based hydrogel matrices are highly biodegradable [23], which could confound measurements. Selecting non-biodegradable or difficult-to-degrade matrices with low cytotoxicity, e.g. polyethyleneglycol-dimethacrylate (PEGDMA), could overcome this issue [24–26]. Co-encapsulation of microbes with particulate C in hydrogels has not been demonstrated yet, despite the potential of this approach as a novel fabricated ecosystem replicating key environmental processes.

Here, we demonstrate a proof-of-concept for hydrogel-based synthetic soil aggregates as an experimental model system. A model particulate C degrading microbe (*Ruminiclostridium cellulolyticum* [27]) was co-encapsulated with cellulose in optically transparent and non-biodegradable PEGDMA hydrogels. Cellulose degradation and growth of *R. cellulolyticum* in these hydrogels was monitored chemically and microscopically. To access information on spatial organization, a staining approach was developed to simultaneously visualize cells, cellulose, and the hydrogel matrix. Overall, this report introduces a reproducible, tailorable experimental model to investigate spatial processes governing C cycling in soil aggregates.

## Materials and Methods

### Cultures and cultivation

*Ruminiclostridium cellulolyticum* H10 ATCC 35319—originally isolated from decayed grass—was used as a model particulate C degrader [27, 28]. *R. cellulolyticum* was routinely grown on a modified VM medium used previously [29]. Briefly, the medium contained (in 1 L): 5.0-g Sigmacell cellulose Type 20, 1.0-g KH_2_PO_4_, 3.4-g K_2_HPO_4_, 2.14-g urea, 1.0-g MgCl_2_.6H_2_O, 0.15-g CaCl_2_.2H_2_O, 1.25-mg FeSO_4_.6H_2_O, 10.0-g 3-(N-morpholino) propanesulfonic acid (MOPS), 1.0-mg resazurin, 1 mL of trace element solution, and 1 mL of vitamin solution. The trace element solution contained (in 1 L), 12.8-g nitrilotriacetic acid, 1.0-g FeCl_2_.4H_2_O, 0.5-g MnCl_2_.4H_2_O, 0.35-g CoCl_2_.6H_2_O, 0.2-g ZnCl_2_, 0.044-g Na_2_MoO_4_.H_2_O, 0.02-g H_3_BO_3_, 0.1-g NiSO_4_.6H_2_O, 0.002-g CuCl_2_.2H_2_O, 0.006-g Na_2_SeO_3_, and 0.008-g Na_2_WO_4_.2H_2_O. The vitamin solution contained (in 100 mL): 2-mg biotin, 2-mg folic acid, 10-mg pyridoxine hydrochloride, 5-mg thiamine hydrochloride, 5-mg riboflavin, 5-mg nicotinic acid, 5-mg DL-pantothenic acid, 5-mg para-aminobenzoic acid, 200-mg choline chloride, and 1-mg vitamin B12. Medium was reduced after autoclaving with a filter-sterilized 0.5% (NH_4_)_2_S-solution to a final concentration of 71-mg (NH_4_)_2_S/L.

### Hydrogel production

**Figure 2:**
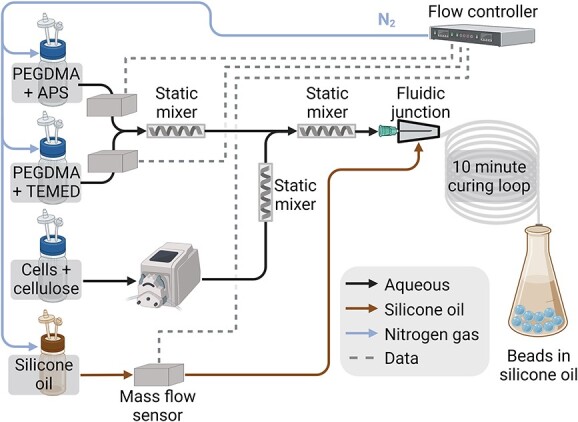
Millifluidic microbe-cellulose-PEGDMA hydrogel production setup. Solutions of PEGDMA monomer with either APS or TEMED are first combined in a static mixer. Then, a suspension of *Ruminiclostridium cellulolyticum cellulolyticum* cells and cellulose is mixed with the polymerizing mixture. Droplets are then formed in a co-flow fluidic junction with silicone oil as carrier fluid. These droplets are allowed to polymerize for 10 minutes in a curing loop, before dropping in a container.

A millifluidic system was developed to simultaneously polymerize the hydrogel matrix and encapsulate a suspension of cellulose and cells (Fig. 2). PEGDMA was chosen as polymer for the hydrogel due to its low biodegradability and low cytotoxicity [24–26]. Nitrogen-sparged PEGDMA solutions (15%) were amended with either ammonium persulfate (APS, 3.64 μM final concentration) or tetramethylethylenediamine (TEMED, 4.93 mM final concentration). An ElveFlow pressure-based millifluidic flow controller (Elvesys, France) driven by N_2_-gas was used to control flow of both solutions at 700 μL·min^−1^, combining them in a low-volume static mixer manually inserted in vinyl tubing. Mixed PEGDMA-monomer and catalysts were then combined in an identical static mixer with a suspension of *R. cellulolyticum* cells and cellulose flowing at 700 μL·min^−1^. Droplets of cell-cellulose-PEGDMA mixture were then formed in a homemade co-flow fluidic junction (1.1-mm orifice size for hydrogel flow) using 10 cSt polydimethylsiloxane polymer (PMX-200 Silicone Fluid, Dow, MI, USA) with 10% cyclopentasiloxane and trimethylsiloxysilicate resin (DOWSIL™ RSN-0749, Dow, MI, USA) as carrier fluid flowing at 1.4 mL·min^−1^. Droplets were allowed to polymerize for 10 minutes in a curing loop (tubing 3-mm inner diameter) before deposition in a flask with flowing N_2_ to prevent O_2_ intrusion needed to protect both bacteria and the polymerization chemistry. Droplets were allowed to fully polymerize into beads overnight before washing.

Bead washing was done inside an anaerobic tent to protect the O_2_-sensitive *R. cellulolyticum*. Beads were first strained over a 400-μm sieve, followed by suspension in carbon-free VM medium at approximately 1:5 bead:liquid volume ratio. Suspensions were centrifuged for 15 minutes at 1000 rcf to separate oil from the aqueous phase. Oil was removed manually before straining beads again. Beads were then washed in carbon-free VM medium +0.1% Tween 80 as bio-compatible detergent (1:5 bead:liquid volume ratio) for 5 minutes. Beads were strained again, followed by three to four washing steps in carbon-free VM medium, until remaining oil was removed.

### Comparing encapsulated and non-encapuslated cellulose degradation

To evaluate the impact of hydrogel encapsulation on *R. cellulolyticum*, cellulose degradation in hydrogels was compared to non-encapsulated growth. Specifically, three conditions were compared: (i) non-encapsulated growth on 15-g cellulose·L^−1^ (CLS), (ii) growth on cellulose in hydrogels (CLS-HG), and (iii) growth on cellulose in hydrogels, but with a 24-hour pre-incubated *R. cellulolyticum* and cellulose suspension (CLS-HG-A) to enable attachment of cells to cellulose particles [27]. Hydrogels were produced from identical broths as the CLS conditions, resulting in a theoretical final cellulose concentration of 5 g·L^−1^ in the hydrogels. All conditions were incubated in 250-mL serum bottles with 50-mL working volume, and an N_2_ headspace. In conditions with hydrogels, 25 mL of the working volume was made up of hydrogels, as measured by volume displacement by beads, while the remaining 25 mL was made up of carbon-free VM medium. Bottles were incubated statically at 37°C. Liquid samples were taken and analyzed for pH. Remaining sample aliquots were in parallel (i) immediately stored at −20°C for total carbohydrate analysis, (ii) diluted 1:1 with 20 mM KOH, filtered over 0.20 μm, and stored at −20°C for organic acids analysis, and (iii) fixed in 4% paraformaldehyde (PFA) for microscopic analysis. Hydrogel samples were taken in the anaerobic chamber, opening them, and sampling 1 mL of beads as measured by volume displacement in a 5 mL cylinder. Some beads were fixed in 4% PFA for microscopic analysis, while the remainder was stored at −20°C for carbohydrate analysis. After sampling, bottles were recapped, and headspace was replaced with N_2_ before continuing incubation.

### Microscopic visualization

The organization of cellulose and cells in the hydrogels were evaluated microscopically. First, hydrogels fixed in PFA were strained and briefly dried. These beads were incubated overnight in Neg-50 sectioning medium (Richard-Allan Scientific, Kalamazoo, USA) at 4°C. Beads were then mounted in custom-made columns with sectioning medium, flash-frozen in a dry ice-ethanol bath for 30 seconds and stored at −80°C until further processing. Individual beads were sectioned into 10-μm sections using a CryoStar NX50 cryotome and mounted on multi-well poly-L-lysine coated slides. For non-encapsulated incubations, 10 μL of 10-fold diluted cell suspension was dried onto poly-L-lysine coated slides at 46°C for 15 minutes.

Samples were stained with SYBR Green nucleic acid stain (cells) and Calcofluor white (cellulose). Specifically, 15 μL of SYBR Green nucleic acid concentrate (10 000x, ThermoFisher, Waltham, MA, USA) diluted 3000-fold in TE-buffer was added per slide well and allowed to incubate in the dark for 30 minutes at 37°C in a moist chamber. After incubation, the SYBR stain was removed manually, and 5 μL of Calcofluor white stain (0.01 g·L^−1^) was added before adding a coverslip. Samples were visualized on a Zeiss Axioskop 2 microscope with mercury arc lamp.

### Analytical methods

Organic acids (C2-C6, including C4 and C5 isoforms) were analyzed with ion chromatography (IC) on a Dionex ICS 5000+ equipped with an IonPac AS11HC analytical and AS11 guard column at 30°C. Eluent consisted of generated KOH (Dionex EG-5 and Dionex EGCIII KOH, Thermo Fisher Scientific) at a flow rate of 1.5 mL·min^−1^. An eluent concentration gradient of 1–60 mM KOH was applied by first running 7 minutes at 1 mM, increasing at (i) 1.56 mM·min^−1^ for 9 minutes, then (ii) 1.67 mM·min^−1^ for 9 minutes, and eventually (iii) 3.75 mM·min^−1^ for 8 minutes up to 60 mM, where concentration was held for 90 seconds. Concentration was then decreased as a step function back to 1-mM KOH for 2 minutes before the end of the run. Component concentrations were externally calibrated (0.5–25 mg·L^−1^) and validated after every 10 samples.

Ethanol was analyzed with HPLC (1260 Infinity II Prime LC, Agilent, CA, USA) equipped with an Aminex HPX-87H column (Bio-Rad, CA, USA). Samples were diluted and pretreated with H_2_SO_4_ at a final concentration of 5 mM. An 80:20 mixture of 5 mM H_2_SO_4_ and acetonitrile as eluent was run isocratically at a flow rate of 0.6 mL·min^−1^. Oven and detector temperatures were kept constant at 30°C. Ethanol was detected on a refractive index detector (RID).

Total carbohydrates were analyzed with the phenol sulfuric acid assay [30]. For hydrogel incubations, hydrogels were first homogenized with a bullet blender (Next Advance, Troy, NY) using 5.6-mm stainless steel UFO beads operated at maximum speed for 15 minutes.

## Results

### Producing and visualizing microbe-cellulose-PEGDMA hydrogel beads

**Figure 3:**
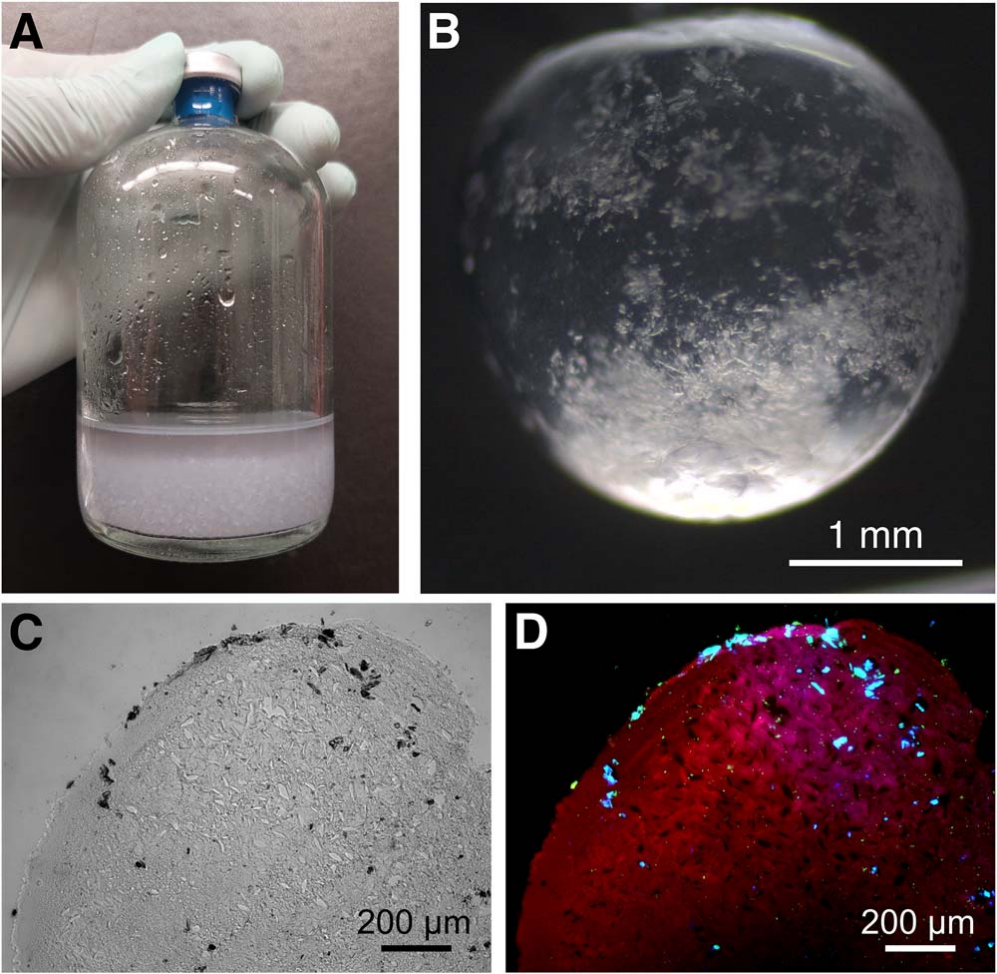
Microbe-cellulose-PEGDMA hydrogel beads. (A) shows hydrogel beads in a 250-mL anaerobic serum flask. (B) shows a magnified hydrogel bead under dissection microscope with cellulose particles visible in the transparent hydrogel matrix. (C) and (D) show a 10-μm-thick hydrogel section under light microscopy and epifluorescent microscopy, respectively. Hydrogels were stained with SYBR green and Calcofluor for visualization of cellulose (blue fluorescence), cellular DNA (green fluorescence), and the hydrogel matrix (red fluorescence).

The millifluidic system produced transparent hydrogels approximately 3 mm in size (note that exact size distribution was not quantifed) with cellulose particles visible within the matrix (Fig. 3A, B). These hydrogels were mechanically stable over long-term incubations and across physical handling (e.g. centrifugation, orbital shaking). A key feature of this experimental model system was the possibility of visualizing cell and cellulose distributions in the hydrogel.

Three methodological steps were integrated to visualize microbes and cellulose particles in hydrogel microsections. First, flash-freezing PFA-fixed hydrogels in a dry ice-ethanol bath was required to preserve the physical structure of the hydrogels during sectioning, and to reliably obtain sections as thin as 10 μm. Second, hydrogel sections had to be deposited on poly-L-lysine coated slides to prevent detachment during subsequent staining steps. Third, a combined SYBR Green-Calcofluor White staining methodology could simultaneously visualize cells (green fluorescence), cellulose (blue fluorescence), and the PEGDMA-hydrogel matrix (red autofluorescence) under epifluorescence microscopy (Fig. 3C, D). This approach provides a simple toolset to investigate spatial dynamics of particulate C degradation in diffusion-limited environments.

### Cellulose degradation and fermentation

To test whether microbial cells survived encapsulation and could access particulate C substrates in hydrogels, the growth of *R. cellulolyticum* on cellulose was compared between (i) conventional, non-hydrogel conditions (CLS), (ii) hydrogel-encapsulated cells and cellulose (CLS-HG), as well as (iii) hydrogel-encapsulated cells and cellulose after a 24-hour pre-incubation (CLS-HG-A).

**Figure 4:**
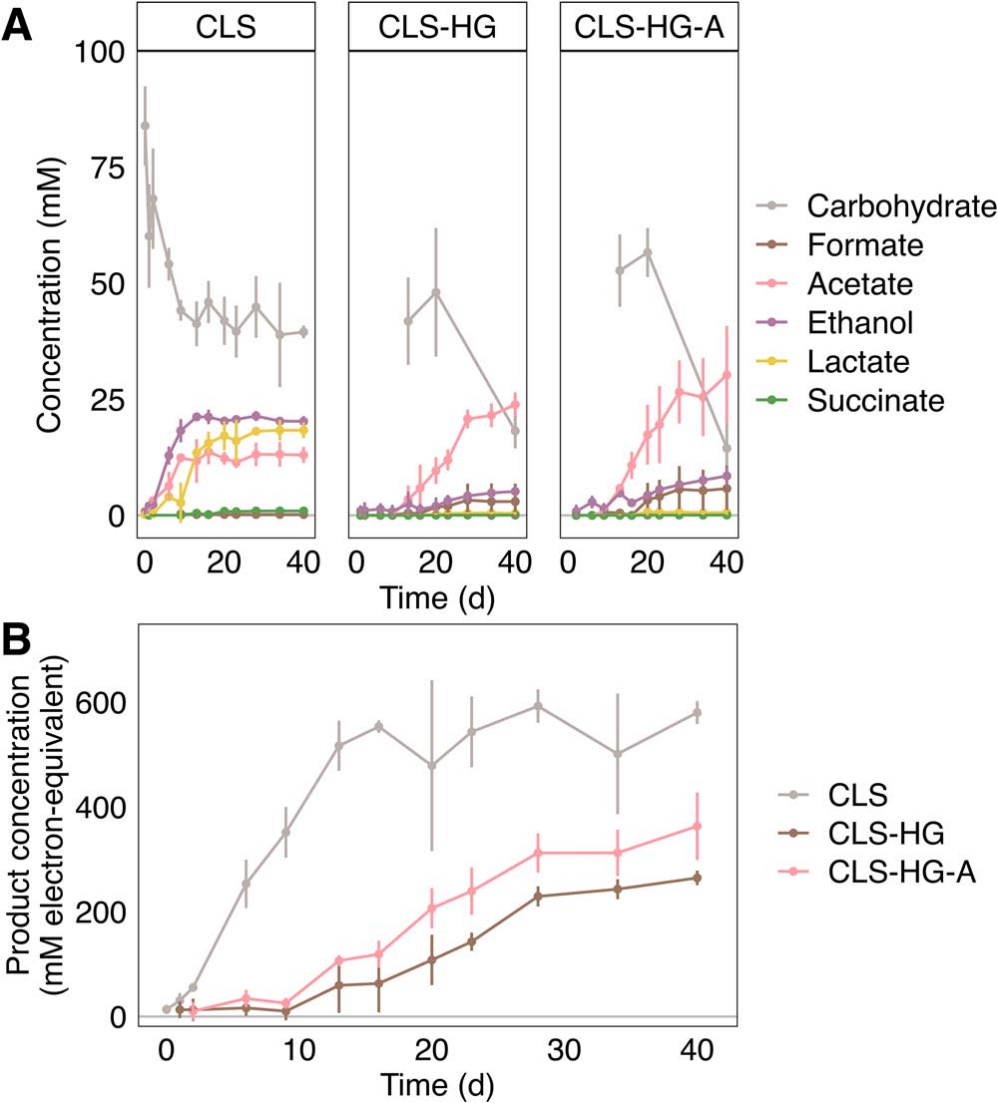
Cellulose fermentation by *R. cellulolyticum* encapsulated in microbe-cellulose-PEGDMA hydrogels. (A) shows the consumption and production profiles over time for *R. cellulolyticum* (i) incubated with cellulose (CLS), (ii) co-encapsulated with cellulose in PEGDMA hydrogels (CLS-HG), and (iii) co-encapsulated with cellulose in PEGDMA hydrogels after a 24-hour incubation (CLS-HG-A). Carbohydrate concentrations are expressed in mM glucose-equivalents. Limited datapoints of hydrogel cellulose-content are available due to the invasiveness of sampling. Note that CLS had an initial cellulose concentration of 15 g·L^−1^, while CLS-HG & CLS-HG-A started at 5 g·L^−1^. (B) shows total product formation over time expressed in electron equivalents for each condition.

Hydrogel conditions had a longer lag time before the start of fermentation (Fig. 4). Cellulose degradation and product accumulation were observed on day 2 in CLS, while product formation was only observed from day 13 in the hydrogel conditions. This indicates the polymerization procedure may have stressed the cells but was not lethal in this study.

Cellulose degradation was observed in both hydrogel and conventional incubations (Fig. 4A), and hydrogel-encapsulation affected total cellulose consumption (ANOVA, *P* = 0.017). Total cellulose consumption in conventional incubations (CLS; 47.2 ± 6.6 mM glucose-equivalents) was significantly higher than in direct hydrogel encapsulation (CLS-HG; 23.6 ± 6.5 mM glucose equivalent; Tukey HSD *P* = 0.014). Total cellulose consumption in pre-incubated hydrogels was not significantly different from either other condition (CLS-HG-A; 38.2 ± 7.8 mM glucose-equivalents; Tukey HSD *P* = 0.33 and 0.09 compared to CLS and CLS-HG, respectively). It should be noted that initial cellulose concentrations differed between conditions (CLS; 15 g·L^−1^, CLS-HG and CLS-HG-A; 5 g·L^−1^) as these were started from the same initial cultures. Controls of CLS incubations at 5-g cellulose·L^−1^ are reported in Supplementary Information (Fig. S1).

Product profiles differed between hydrogel and conventional incubations (Fig. 4A). The dominant products of conventional cellulose degradation were ethanol (20.2 ± 1.1 mM), lactate (18.3 ± 1.7 mM), and acetate (13.0 ± 1.7 mM). Hydrogel-encapsulated conditions, on the other hand, predominantly produced acetate (CLS-HG, 23.8 ± 2.6 mM; CLS-HG-A, 30.2 ± 1.1 mM), with lower concentrations of ethanol (CLS-HG, 5.2 ± 1.2 mM; CLS-HG-A, 8.5 ± 2.0 mM) and formate (CLS-HG, 3.0 ± 3.8 mM; CLS-HG-A, 5.8 ± 5.0 mM), while lactate remained below 1 mM.

Total product formation was influenced by hydrogel encapsulation (Fig. 4B). Conventional cellulose incubations generated most product (CLS: 580.1 ± 21.7 mM e^-^-equivalents), followed by pre-incubated cellulose in hydrogels (CLS-HG-A: 363.3 ± 64.4 mM e^-^-equivalents) and directly encapsulated cellulose in hydrogels (CLS-HG: 264 ± 13.8 mM e^-^-equivalents). Total product formation was significantly different between conditions (ANOVA, *P* = 0.0002). However, this difference was only significant between non-hydrogel and hydrogel conditions (Tukey HSD testing, CLS and CLS-HG, *P* < 0.001; CLS & CLS-HG-A, *P* = 0.002), while no significant difference was found between the two hydrogel conditions (Tukey HSD testing, CLS-HG and CLS-HG-A, *P* = 0.053).

Overall, hydrogel encapsulation was shown to enable growth of *R. cellulolyticum* on particulate cellulose, although this approach affected lag time and fermentation product profiles.

### Monitoring spatial organization in microbe-cellulose-PEGDMA hydrogels

**Figure 5:**
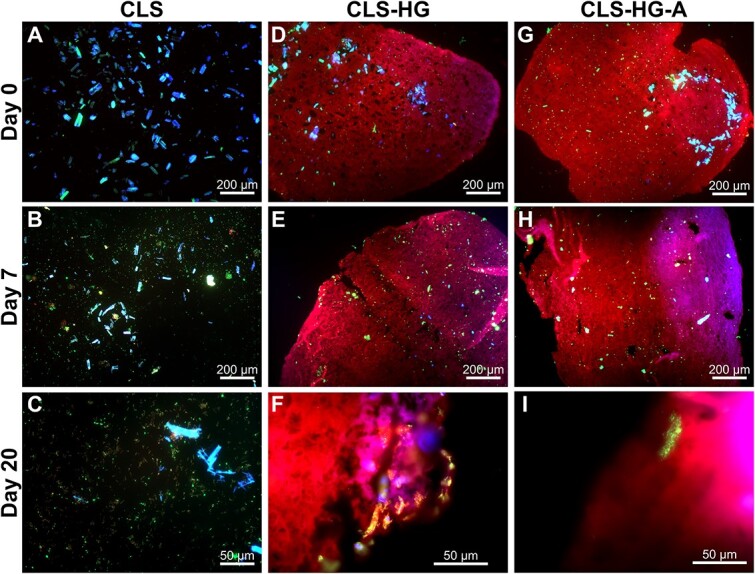
Visualizing *R. cellulolyticum* cells and cellulose particles with fluorescence microscopy. Cellulose (blue fluorescence), cells (green fluorescence), and hydrogel matrix (red fluorescence) are shown in conventional cellulose incubations (CLS; A−C), hydrogel-encapsulated cellulose (CLS-HG; D−F) and pre-incubated hydrogel-encapsulated cellulose (CLS-HG-A; G−I). (A), (D), and (G) represent the start of the incubation, (B), (E), and (H) represent the midway point (i.e. day 7 for CLS, day 20 for CLS-HG and CLS-HG-A), and (C), (F), and (I) represent the same timepoint at higher magnification.

Scale-bridging experimental models of soil aggregates should enable visualizing spatial assembly of microbial communities. Here, the modified experimental approaches for microscopic evaluation of hydrogels (Fig. 3) enabled qualitative observation of cellulose degradation and cell growth in both conventional (CLS, Fig. 5A−C) and hydrogel (CLS-HG, Fig. 5D−F; CLS-HG-A, Fig. 5G−I) incubations. Across all conditions, an increase in green fluorescence (i.e. cells) and decrease in blue fluorescence (i.e. cellulose) was observed, although not quantified. Additionally, individual cells could be observed around cellulose particles in CLS-HG and CLS-HG-A (Fig. 5F and I, respectively). Such organizations could not be seen in CLS, and cells appeared more dispersed between residual cellulose particles (Fig. 5C). This proof-of-concept demonstrates that it is possible to visualize solid carbon degradation and cell growth in hydrogels as a reproducible model for diffusion-limited systems in environmental and engineered settings.

## Discussion

### Microbe-cellulose-PEGDMA hydrogels sustain growth of particulate C degraders

This study demonstrates (i) co-encapsulation of particulate C substrates and hydrolytic microbes in hydrogels, (ii) monitoring of cellulose fermentation in these hydrogels, and (iii) visualisation of microbial growth around hydrogel-encapsulated cellulose particles.

Hydrogel encapsulation of microbes is a commonly used technique to develop novel biotechnological consortia or investigate fundamental ecological interactions. For instance, pairing nitrogen-cycling microbes not typically found together in water treatment facilities supports low-energy water treatment technologies [21, 31], while hydrogel-encapsulated gut microbiota have been used to investigate the role of spatial organization in gut microbiomes [32]. Hydrogel-modification for enhanced cultivation has, so far, mostly emphasized polymer and crosslinking chemistry [33–35], whereas the addition of functional particulates has remained sparse. Recent examples include the addition of conductive particles to hydrogels to support the growth of electroactive microbes [36, 37], and the addition of carbon black particles to enable selective biomass heating for low-temperature applications [38]. While these examples add functional elements to support microbial activity, the microbe-cellulose-PEGDMA hydrogel system developed here is the first to encapsulate cells along with particulate, catabolic substrates (Figs 3 and 5).

Hydrogel encapsulation shifted the product profile generated by cellulose-degrading *R. cellulolyticum*. In non-hydrogel incubations, *R. cellulolyticum* produced a mixture of ethanol, lactate, and acetate from cellulose, while this shifted to predominantly acetate, with lower concentrations of ethanol and formate in both hydrogel-encapsulated conditions. Previous work has shown initial cellulose concentrations controls product profiles of *R. cellulolyticum* in batch incubations, with more cellulose resulting in higher lactate concentrations and a maximum of ethanol and acetate at 6.7-g cellulose·L^−1^ [39]. While the absence of lactate in CLS-HG and CLS-HG-A could be attributed to lower cellulose concentrations, it should be noted that a 5-g cellulose·L^−1^ incubation - equivalent to the hydrogel conditions - yielded more lactate than either hydrogel condition (Fig. S1), an observation also mirrored in literature data [39]. Similarly, ethanol concentrations in conventional incubations always exceed acetate concentrations, regardless of initial cellulose concentration [39], while CLS-HG and CLS-HG-A here showed an acetate-dominated product profile (Fig. 4A). It is worth noting that the obtained product profiles in the hydrogel conditions are quite similar to those obtained in continuous cultivation of *R. cellulolyticum* [40, 41]. This altered energy metabolism could be due to substrate and product diffusion gradients in the hydrogels, or due to phenotypic or regulatory changes in response to physical confinement [42, 43], although these hypotheses remain to be tested. Overall, incubation of *R. cellulolyticum* in microbe-cellulose-PEGDMA hydrogels resulted in a more selective and more oxidized (i.e. acetate) product profile compared to conventional incubations.

Hydrogel encapsulation induced a lag phase in *R. cellulolyticum* activity. While some hydrogel polymers can be toxic to cells, PEGDMA has been shown to exert low toxicity to cells [25, 26]. We therefore hypothesize the lag phase may be due to stress during hydrogel crosslinking or due to encapsulation as such. Crosslinking of PEGDMA requires free radicals, in this case generated by APS and TEMED, which may stress cells [44]. Alternatively, encapsulation in hydrogels may induce an initial lag phase as has been observed in polyvinyl alcohol-sodium alginate hydrogels previously, although even fastidious ammonium oxidizing archaea have been shown to overcome this lag phase [20, 45]. This encapsulation stress could be overcome by *R. cellulolyticum*, yet, how this extends to other organisms and complex communities remains to be investigated.

Pre-attachment of cells to cellulose particles did not improve cellulose degradation or product formation. *Ruminiclostridium cellulolyticum* physically attaches to cellulose particles for degradation and fermentation [27]. Given that these processes were not significantly changed between direct and pre-attached encapsulation (Fig. 4), this suggests that cells may be able to move through the hydrogel matrix. Regardless of the cell mobility, these results indicate that cellulose is an easily accessible substrate in PEGDMA matrices.

Overall, this study demonstrates for the first time the possibility of incubating microbes on particulate C substrates in microbe-cellulose-PEGDMA hydrogels and that hydrogel encapsulation may modulate organisms’ metabolism.

### Hydrogel-based fabricated ecosystems

Fabricated ecosystems for soil aggregates should replicate key processes at the micro-to-millimeter scale controlling microbial activities; (i) diffusion gradients of e.g. O_2_ or NO_3_^−^ in soil aggregates [4, 46], (ii) soil aggregate pore structure, which spatially limits diffusion, making micropores (<50 μm) important niches for soil microbes [7], and (iii) particulate C embedded in soil aggregates creating hotspots of microbial activity [7]. The microbe-cellulose-PEGDMA hydrogels developed here can capture these key processes. First, hydrogels are well-described to be diffusion-limiting environments sustaining chemical gradients [21, 47]. A theoretical calculation estimates diffusivity of O_2_ in the PEGDMA-hydrogels is approximately 1500 μm^2^·s^−1^, which lies between diffusivity of O_2_ in pure water (2500 μm^2^·s^−1^) and that in millimeter-scale soil aggregates (960 μm^2^·s^−1^) [48, 49]. Second, PEGDMA hydrogels produced here appear to have sufficient porosity to enable cell mobility, as no significant difference was observed between CLS-HG and CLS-HG-A conditions. Last, the inclusion of particulate C supported the growth of *R. cellulolyticum*, which could be tracked microscopically (Fig. 5). Consequently, we propose the microbe-cellulose-PEGDMA hydrogels are suited to become a standardizable experimental model - or, fabricated ecosystem [11] - for soil aggregate studies.

Some limitations of the current microbe-cellulose-PEGDMA hydrogel system should be overcome in future research. For instance, cellulose clusters together to one side of the hydrogel (Figs 3 and 5) indicating work is needed to distribute cellulose more homogeneously in the matrix with optimized millifluidic approaches. This heterogeneous cellulose distribution currently limits the use of quantitative microscopic analyses, despite their potential to improve our understanding of the system. Visualizing heterogeneous distributions with sections means a vast amount of sections would be needed to reliably quantify spatial dynamics. However, if future applications can homogenize cellulose distribution, integrating FISH could be a powerful tool to evaluate community assembly around particulate C across intra-aggregate chemical gradients. Hydrogel architecture - including aggregate size, porosity and pore size distribution - is another factor to optimize in future iterations. Despite the apparent mobility of cells in the hydrogel matrix, replicating soil aggregate architectures will require measuring and quantifying hydrogel porosity and pore size distributions. Future work could aim to modify hydrogel formulations and production procedures to control bead sizes as well as introduce larger pores and control pore sizes in the hydrogels to more realistically simulate soil aggregate pore structure and investigate its impact on microbial metabolism [7, 50, 51]. Last, the current hydrogel system was only evaluated under fully hydrated conditions. While this could make the system valid for wetland ecosystems [6], further research should explore how to leverage this model to investigate drought events and unsaturated upland soils. Alternatively, other types of gel matrices, such as dry xerogels [52], may be useful in replicating upland soil aggregates. Overcoming these limitations relating to gel morphology, analysis and experimental modulation could make this hydrogel-based experimental model more widely applicable.

The microbe-particulate C-hydrogel system is well-positioned as a standardizable fabricated ecosystem offering key opportunities to answer critical research questions for environmental microbiologists. This proof-of-concept encapsulated a single organism (*R. cellulolyticum*) and a simple particulate C substrate (cellulose). The synthetic nature of these model soil aggregates allows researchers to tailor what is encapsulated in the hydrogels to the research question at hand. For instance, encapsulation of synthetic microbial communities replicating environmental microbiomes in these hydrogels would provide a highly tractable system of particulate C degradation and its responses to environmental stressors [11, 53, 54]. Encapsulating soil microbiomes could further replicate more complex natural processes while maintaining a higher degree of experimental controllability compared to *in situ* or *ex situ* soil studies. Additionally, the particulate C source can also be tailored from simple (e.g. cellulose) to more complex (e.g. particulate plant biomass) to cover a range of biological complexity. Critically, the low-biodegradability of the hydrogel matrix prevents issues deconvoluting microbial hydrogel degradation from particulate C degradation [24]. The millimeter-scale synthetic soil aggregates are also ideally poised to bridge scales. Evaluating hydrogel-encapsulated microbes with FISH-nanoSIMS for single-cell identity and activity analysis would effectively connect individual cells to millimeter-scale process outcomes [55]. Moreover, this model could be further extended to meter-scale soil columns by assembling these synthetic soil aggregates in bioreactors with controlled environmental parameters (e.g. T, salinity, O_2_, etc.). In this way, the microbe-particulate C-PEGDMA hydrogels developed here could form the basis of fabricated ecosystems connecting individual soil microbes to ecosystem-scale process outcomes.

This study develops a novel experimental system that co-encapsulates microbes (here, *R. cellulolyticum*) and particulate C in hydrogels. These hydrogels supported cellulose fermentation and induced a shift in the metabolism and product profile of *R. cellulolyticum*. Microscopic visualization demonstrated the possibility to track cellulose degradation and cell growth in spatially explicit systems. These microbe-cellulose-PEGDMA hydrogels provide a tailorable, reproducible fabricated ecosystem to study particulate C degradation in soil aggregates that could be used to bridge activities and distributions of microbes to soil aggregate and ecosystem-scale process outcomes.

## Supplementary Material

Candry_et_al-CelluloseHydrogels_SI_ycae068

## Data Availability

Data is available from the corresponding author upon reasonable request.
