# Supplementary material for: Microbe-cellulose hydrogels as a model system for particulate carbon degradation in soil aggregates
Source: ISME Commun. 2024 May 4;4(1):ycae068. doi: 10.1093/ismeco/ycae068 (PMC11126157; doi:10.1093/ismeco/ycae068)
Supplement: Candry_et_al-CelluloseHydrogels_SI_ycae068 [file candry_et_al-cellulosehydrogels_si_ycae068.pdf]

SUPPLEMENTARY INFORMATION

Supplementary Information to: Microbe-cellulose-PEGDMA hydrogels as an experimental model for particulate carbon degradation in soil aggregates

Running Title: Hydrogel-based synthetic soil aggregates

Pieter Candry[1,2,#], Bruce J. Godfrey[1], Mari Karoliina-Henriikka Winkler[1]

[1] Civil and Environmental Engineering, University of Washington, 201 More Hall, Box 352700, Seattle, WA 98195-2700, USA

[2] Laboratory of Systems and Synthetic Biology, Wageningen University & Research. 6708 WE, Wageningen, The Netherlands

[#] Correspondence to: Pieter Candry, Civil and Environmental Engineering, University of Washington, 201 More Hall, Box 352700, Seattle, WA 98195-2700, USA; E-mail: pcandry@uw.edu

* Current Address: Laboratory of Systems and Synthetic Biology, Wageningen University & Research. 6708 WE, Wageningen, The Netherlands; E-mail: pieter.candry@wur.nl

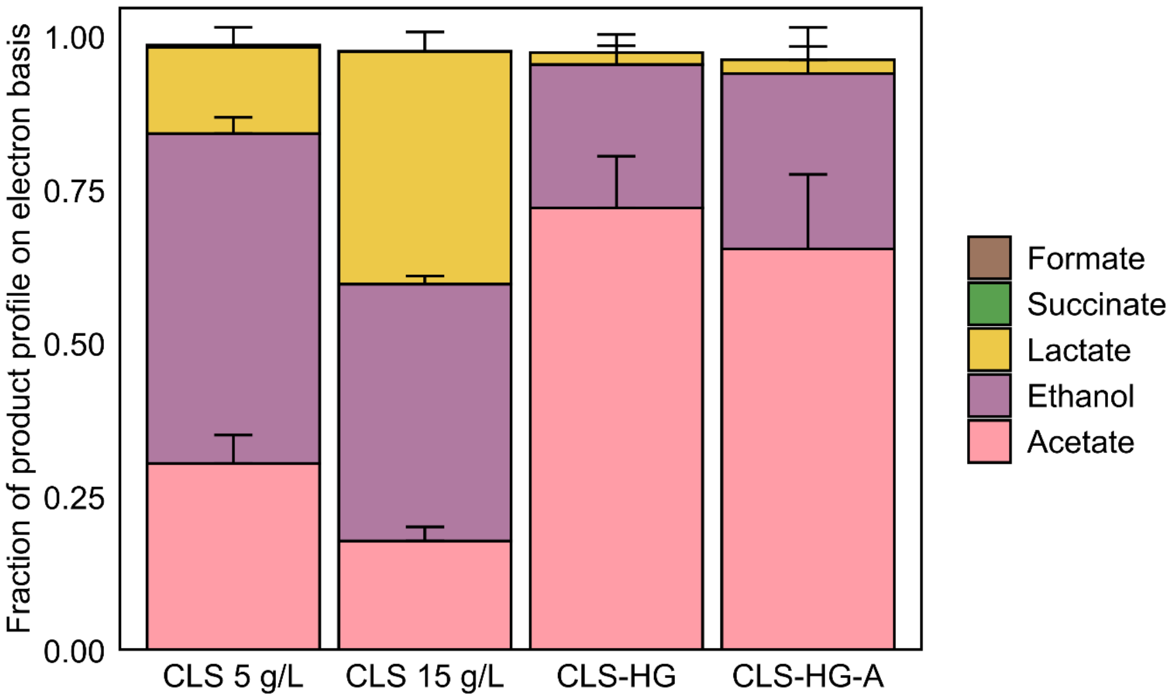

Figure S.1. Impact of hydrogel encapsulation on fermentation product profiles. Product profiles are compared between conventional incubations at 2 cellulose concentrations (5 and 15 g cellulose·L^{-1}) and hydrogel-encapsulated incubations at a theoretical initial cellulose concentration of 5 g cellulose·L^{-1}.
